# Preliminary study on hand dimensions as potential predictors of female populations native to forest and savanna zones in Ghana

**DOI:** 10.1038/s41598-024-59403-x

**Published:** 2024-09-27

**Authors:** Samuel Bimpong, Chrissie Stansie Abaidoo, Joshua Tetteh, Francis Kofi Sarkodie, Collins Adjei-Antwi, James Nketsiah, Atta Kusi Appiah, Thomas K. Diby

**Affiliations:** Department of Anatomy, School of Medicine and Dentistry, Kwame Nkrumah University of Science and Technology, Kumasi, Ghana

**Keywords:** Preliminary, Ethnicity, Natives, Forest zone, Savanna zone, Hand dimension, Biological techniques, Cell biology, Genetics, Structural biology, Climate sciences, Anatomy, Health occupations, Medical research

## Abstract

The hand is a versatile structure that performs numerous tasks, ranging from exertion of great force such as grip, pinch and torque to execution of precise fine motor skills. The aim of current study was to undertake a preliminary study on hand dimensions as potential predictors of female populations native to the forest and savanna zones of Ghana. A total of one hundred (100) female students aged between 17 and 24 years were recruited into this study, comprising of 53 native to the forest zone and 47 native to savanna zone of Ghana between 12th June to 27th July, 2023 at the Department of Anatomy, School of Medicine and Dentistry, Kwame Nkrumah University of Science and Technology. Statistically significant positive correlation was observed between left hand length and right hand length (R = 0.923, p = 0.000). From the binary regression model, it could be speculated that left-hand breadth could predict female populations native to the savanna zone (LHB: β = − 2.37, Expβ = 0.09, p = 0.014). However, right-hand breadth and length and left hand length did not show any potential of prediction (RHB: β = 0.900, Expβ = 2.460, p = 0.410; RHL: β = 0.168, Expβ = 1.683, p = 0.803; LHL: β = − 0.300, Expβ = 0.741, p = 0.656). The study therefore may speculate that left handbreadth could have the potential to differentiate female populations native to savanna zone from females native to forest zone in Ghana.

## Introduction

Hand is the distal region immediately after the forearm of the upper extremity. It mostly discharges the functions which the brain wants to perform physically as is evident in the use of tools invented by man’s brain. The skeleton of the hand consists of eight carpal and five metacarpal bones as well as fourteen phalanges^1^. The function of the hand involves combined complex interaction between the force generated by the intrinsic and extrinsic musculature, stable position offered by ligaments and structural support provided by the bones^2^.

Scientific significance of studying human hand have been found in fields such as anthropometry, forensic pathology, orthopaedic surgery and ergonomics^3^. Also, anthropometric data on hand dimensions have been used in resolving legal, medical and forensic cases^4^. Race and ethnicity are closely related terminologies, yet a sharp contrast exists between the two terms; “race” is based upon criteria which in the past were assumed to be biologically inherited within a group, while ‘ethnicity” makes reference to a population whose membership identity is grounded on the basis of common heritage, culture and language. Hence there could be various ethnicities in a particular race that may differ in terms of geographical location, culture, economic activities, nutritional requirements etc.^5,6^. According to a study, the various ethnic groups in Ghana are classified under three major climatic zones namely the forest zone, coastal zone and savanna zone^7^.

Owing to the crucial role played by anthropometric data in ergonomics, security, health and industries, many countries have made strenuous efforts to establish anthropometric database of their populations using various body measurements. These studies mostly employed the measured anthropometrics to determine stature, sex, race and ethnic groups. Hand dimensions have been used in many of these studies^5,8–12^. Also, race and ethnicity influence divergence in hand dimensions leading to different values observed in men and women^13^. In Ghana, there appears to be dearth of information on anthropometric measurements and ethnicity. A recent study that sought to use body anthropometry to identify the ethnicity of three major ethnic groups in Ghana, used the foot dimensions^14^. It is therefore, the aim of this study to undertake preliminary study on hand dimensions as potential predictors of female populations native to the forest and savanna zones in Ghana.

## Materials and methods

This was a cross sectional study carried out at the Department of Anatomy, School of Medicine and Dentistry, Kwame Nkrumah University of Science and Technology (KNUST). KNUST community offers a unique opportunity of having students from various climatic zones.

A total of one hundred (100) female students aged between 17 and 24 years were recruited into this study, comprising of 53 females native to forest zone and 47 native to the savanna zone of Ghana. The study adopted the forest and savanna zones because Females native to forest zone speaking people dominate the forest zone while Hausa, Dabgani, Kusaal, Frafra, Dagaare etc. are spoken by the people in the savanna zone. Since this was a preliminary study, sample size was determined using sample size guidelines for logistic regression rule of thumb for the minimum sample size based on event per variable (EPV) of 10^15^. Data collection spanned 12th June to 27th August, 2023.

Inclusion into the study was based on female students who were native to any of the two climatic zoness, who consented to be enrolled and without any physical deformity of the left and right hands. Male, and foreign students, female students with deformed hands and those who did not consent to participate in the study were excluded from this study. Ethical clearance was sought from the Committee on Human Research, Publication and Ethics (CHRPE). Informed consent of participants was sought prior to the study. The study methods were performed in accordance with guidelines and regulations of the CHRPE.

### Demographic characteristics

Participants age and climatic zone were recorded against their individual unique identification numbers in the field book.

### Hand length measurement

**Figure 1:**
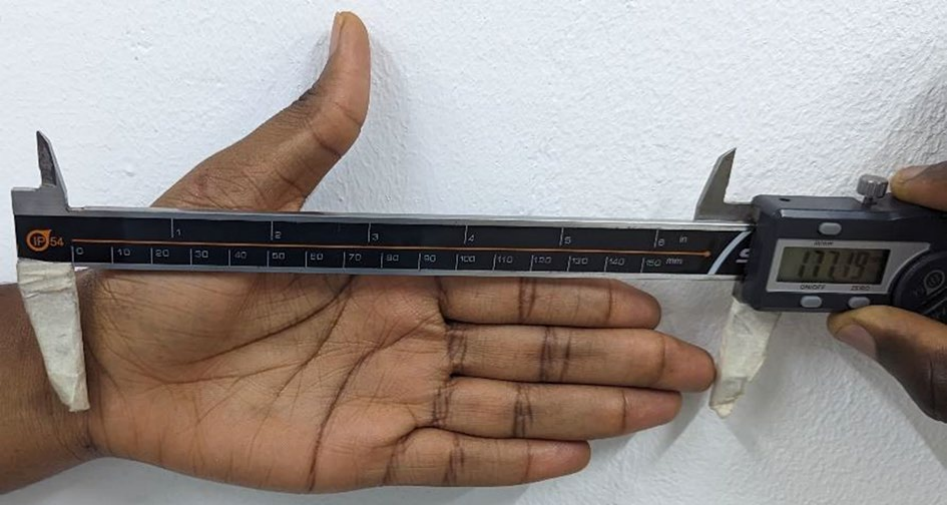
Measurement of hand length.

Hand length was measured using digital sliding Venier calipers (SHAHE 0–500 mm Digital Vernier caliper. Wenzhou Sanhe Measuring Instrument Co. Ltd), as a distance between the distal wrist crease (Mid-stylion) to the tip of the middle finger (Dactylion). Participants were made to extend the hand in supine position, with their fingers fully extended. The end pointer of the caliper was placed at the mid-stylion line and the housing pointer applied to the dactylion. An average value was then calculated from three separate measurements (Fig. 1).

### Hand breadth measurement

**Figure 2:**
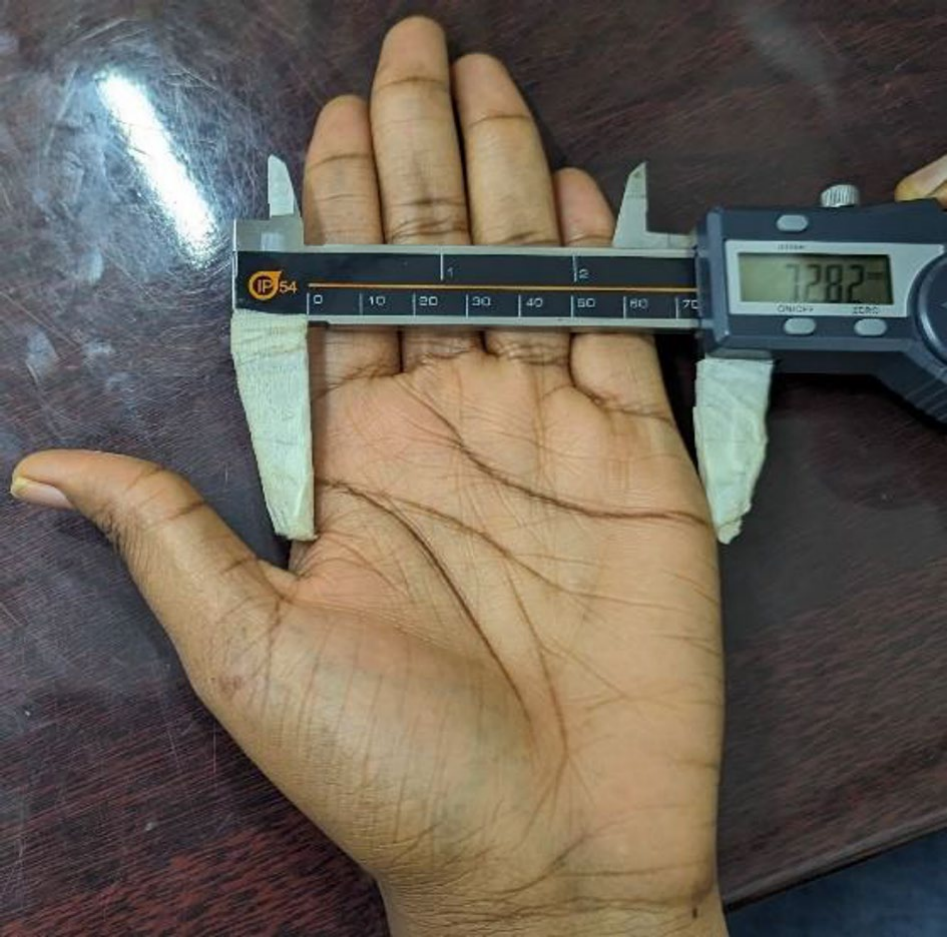
Hand breadth measurement.

This was determined as the distance between the most prominence on the lateral part of the head of second metacarpal and most prominence projection on the medial region of the head of fifth metacarpal. Participants were asked to raise their hands in a supine position with the palm and fingers stretched firmly (Fig. 2). The caliper was inclined at 45° to palpate the landmarks and measurements were taken in millimeters which was later converted into centimeters.

### Statistical analysis

Statistical analysis was performed using IBM Statistical Package for Social Sciences (IBM SPSS Statistics. Version 26.0. Chicago Inc.). Measurements were presented in descriptive table (mean, standard deviation, maximum and minimum). Unpaired sample t test was used to compare all hand dimensions in females native to the two climatic zones to determine statistical significance of mean differences of all hand dimensions. Statistical significance was at p < 0.05. Binary logistic regression model was derived to predict female population native to forest or savanna zone using hand dimensions.

### Ethical approval

Prior to the study, ethical approval was obtained from the Committee on Ethics and Human Research for Publication, School of Medicine and Dentistry, Kwame Nkrumah University of Science and Technology. Kumasi with approval number CHRE/AP/128/23. Ghana. Informed Consent was obtained from the study participants. The study methods were in accordance with the guidelines and regulations of CHRPE–KNUST.

## Results

### Descriptive statistics of study participants’ age and hand dimensions

The mean age of the total participants was 19.15 ± 1.20 years, similarly, the mean age of the females native to forest zone was 19.15 ± 1.28 years. Mean age for the females native to savanna zone was 19.15 ± 1.22 years. The mean left hand breadth of the total sample was 7.78 ± 0.38 cm. The mean left hand breadth of the females native to forest zone was 7.66 ± 0.37 cm while females native to savanna zone had mean left hand breadth of 7.90 ± 0.35 cm. The mean left hand length for the total sample was 17.68 ± 0.83 cm. Females native to forest zone had mean left hand length of 17.55 ± 0.83 cm and females native to savanna zone had 17.83 ± 0.80 cm. Right hand breadth of the total sample was 7.85 ± 0.31 cm. The mean right hand breadth of the females native to forest zone was 7.79 ± 0.32 cm, females native to savanna zone had 7.91 ± 0.28 cm as the mean right hand breadth. Right hand length mean of the total group was 17.63 ± 0.82 cm. Mean right hand lengths of 17.50 ± 0.81 cm, and 17.77 ± 0.82 cm, were recorded for females native to forest zone and females native to savanna zone respectively, (Table 1).

**Table 1:** Descriptive statistics of study participants’ age and hand dimensions.

Variable	Total		Females native to forest zone		Females native to savanna zone	
	Mean ± SD	(Range)	Mean ± SD	(Range)	Mean ± SD	(Range)
Age (years)	19.15 ± 1.20	(17–24)	19.15 ± 1.28	(17–24)	19.15 ± 1.22	(17–22)
LHB (cm)	7.78 ± 0.38	(6.98–8.82)	7.66 ± 0.37	(6.98–8.82)	7.90 ± 0.35	(7.19–8.51)
LHL (cm)	17.68 ± 0.83	(16.01–20.13)	17.55 ± 0.83	(16.01–19.65)	17.83 ± 0.80	(16.43–20.13)
RHB (cm)	7.85 ± 0.31	(7.28–8.75)	7.79 ± 0.32	(7.28–8.64)	7.91 ± 0.28	(7.34–8.75)
RHL (cm)	17.63 ± 0.82	(16.09–20.04)	17.50 ± 0.81	(16.09–19.68)	17.77 ± 0.82	(16.39–20.04)

LHB: left hand breath; LHL: left hand length; RHB: right hand breath; RHL: right hand length; SD: standard deviation.

Pearson’s correlation was performed between the hand dimensions of the total population to establish relationships among the various hand dimensions of both left and right hands in Table 2. Strong positive correlations were observed between the left hand breadth and length (R = 0.437, p < 0.001), left hand breadth and right hand breadth (R = 0.751, p < 0.001) and left hand breadth with right hand length (R = 0.442, p < 0.001). While left hand length positively correlated with right hand breadth (R = 0.426, p < 0.001). Correlation between left hand length and right hand length was strongly positive (R = 0.923, p ¸0.001). Between right hand breadth and right hand length, the correlation was also positive (R = 0.414, p < 0.001).

**Table 2:** Pearson correlations between hand indices of study participants.

Variables		Left hand breadth	Left hand length	Right hand breadth	Right hand length
Left hand breadth	R	1.000	0.437	0.751	0.442
	p-value		< 0.001	< 0.001	< 0.001
Left hand length	R		1.000	0.426	0.923
	p-value			< 0.001	< 0.001
Right hand breadth	R			1.000	0.414
	p-value				< 0.001
Right hand length	R				1.000
	p-value				

R: coefficient of correlation, p-value: statistical significance at p < 0.01.

This present study also compared the age and hand dimensions between the two zones in Table 3. There was no statistically significance in the mean age of the two groups. However, significant difference was observed between the mean values of left hand breadth measurement in the two female populations (p = 0.001). Although, the mean value of left hand length was higher for the females native to savanna zone than the females native to forest zone counterpart, the difference was not significant statistically (P = 0.060). The mean value of right hand breadth measurement was higher in the females native to savanna zone than the females native to forest zone. This showed statistical significance with p = 0.037 while right hand length though, higher in the females native to savanna zone population than in females native to forest zone, the difference was not significant (p = 0.099).

**Table 3:** Comparison of age and hand indices between females native to forest zone and females native to savanna zone.

Variable	Females native to forest zone	Females native to savanna zone	p-value
Age (years)	19.15 ± 1.28	19.15 ± 1.22	1.000
LHB (cm)	7.66 ± 0.37	7.90 ± 0.35	**0.001**
LHL (cm)	17.55 ± 0.83	17.83 ± 0.80	0.060
RHB (cm)	7.79 ± 0.32	7.91 ± 0.28	**0.037**
RHL (cm)	17.50 ± 0.81	17.77 ± 0.82	0.099

LHB: left hand breath; LHL: left hand length; RHB: right hand breath; RHL: right hand length; SD: standard deviation.

Significant values are in bold.

Binary logistic regression was performed to ascertain the likelihood of left and right hand dimensions predicting the females native to forest zone as against those native to savanna zone. From Table 4, The binary regression model showed that right hand breadth and length as well as left hand length could not predict female populations native to forest zone (RHB: β = 0.900, Expβ = 2.460, p = 0.410; RHL: β = 0.168, Expβ = 1.683, p = 0.803; LHL: β = − 0.300, Expβ = 0.741, p = 0.656), however, left hand breadth showed potential of predicting female population native of savanna zone with statistical significant level (LHB: β = − 2.37, Expβ = 0.09, p = 0.014).

**Table 4:** Components of binary logistic regression model using hand dimensions to predict females native to forest zone.

Hand dimensions	B	S.E.	Wald	Df	p-value	Exp(B)	95% C.I. for EXP(B)	
							Lower	Upper
LHB	− 2.370	0.969	5.987	1	0.014	0.093	0.014	0.624
LHL	− 0.300	0.673	0.198	1	0.656	0.741	0.198	2.773
RHB	0.900	1.092	0.679	1	0.410	2.460	0.289	20.928
RHL	0.168	0.674	0.062	1	0.803	1.183	0.316	4.432
Constant	13.832	6.278	4.854	1	0.028	1,016,680.459		

LHB: left hand breadth; LHL: left hand length; RHB: right hand breadth; RHL: right hand length; B: beta coefficient; SE: standard error; Wald: Wald statistic; Df: degree of freedom; p-value: significant level < 0.05; Exp(B): odds ratio; 95% C.I: 95% confidence interval. Constructed model is Ethnicity = β × hand parameter(s) + constant, the cut-up value is 0.5 (that is predictive probability is for Females native to forest zone ethnicity), Value ≤ 0.5 is indicative of Northerner Ethnicity and value > 0.5 is indicative of Females native to forest zone ethnicity.

Table 5 below illustrates the prediction accuracy and the overall percentage accuracy of classifying ethnicity. Numerous inconsistencies existed in classifying the Northern ethnicity, but was more consistency in the Females native to forest zone ethnic group. Left hand breadth had the highest overall percentage accuracy rate of 66.0% while left hand length scored the lowest overall percentage accuracy of 54%. Right hand breadth was next to left hand breadth in terms of the percentage accuracy rate (62%), and right hand length percentage accuracy rate (56%) was higher than left hand length.

**Table 5:** Prediction and percentage accuracy of zonal discrimination by hand length and breadth.

Hand dimensions	Zone	Prediction accuracy		%Accuracy
		Savanna natives	Forest natives	
LHB	Females native to savanna zone	27	20	57.4
	Females native to forest zone	14	39	73.6
	Overall % accuracy			66.0
LHL	Females native to savanna zone	16	31	34.0
	Females native to forest zone	15	38	71.7
	Overall % accuracy			54.0
RHB	Females native to savanna zone	21	26	44.7
	Females native to forest zone	12	41	77.4
	Overall % accuracy			62.0
RHL	Females native to savanna zone	17	30	36.2
	Females native to forest zone	14	39	73.6
	Overall % accuracy			56.0

LHB: left hand breadth; LHL: left hand length; RHB: right hand breadth; RHL: right hand length.

## Discussion

This current study was conducted on female populations from two major ethnic groups, the Females native to forest zone and Females native to savanna zone which dominate the rest of ethnic groups in the forest and savanna zones of Ghana respectively. The age range of study participants in the present study was 17–24 years. The mean age of the females native to forest zone and Females native to savanna zone were similar to that of the general population.

The current hand breadths mean values were slightly higher than those recorded for female populations from four different ethnic groups in Malaysia, namely Kadazandusun, Bajau, Malay and Chinese^16^. The findings of the present study were again, higher than the mean values observed for three different ethnic groups also from Malaysia^17^. However, the present hand breadths values were lower than a counterpart study conducted in Nigeria among the Delta Igbos and Isokos^10^. These variations could probably result from genetics, cultural, nutritional, economics or geographical factors.

In this present study, the mean left and right hand lengths values for the total population, the females native to forest zone, females native to savanna zone were higher than the mean values recorded for the Kadazandusun,, Bajau, Malay and Chinese^16^ and in the Malay, Chinese and Indians^17^. On the contrary, hand length (left and right) measurements in this current study were lower than those observed for the Delta Igbos and Isokos in Nigeria^10^. Another study in India also recorded slightly lower left and right hands dimensions as compared to this present study values in the female population. Also, it was observed that left hand dimensions were slight lower than that of right hand values and this has been reported in a study of Kashmiri Pandits and Haryanvi Brahmins ethnic groups in India^18^. The observation affirms cross-cultural, geographical, and economic variations in hand anthropometry. It also, shows the existence of bilateral asymmetry in the populations involved in these studies.

The results of Pearson’s correlation demonstrated statistically significant and strong positive correlations between the left and right hand breadths and between left and right hand lengths. The correlations between the hand breadths and lengths though, positive but were not as strong as were observed within hand breadths or lengths (Table 2). Though the correlation coefficients observed in the present study were a little higher than recorded in a similar study in India for female population, the results of both studies were in agreement^19^. The findings were also consistent with the study results reported in hand anthropometric data of Saudi Arabian students^20^.

This study compared the hand dimensions of the two ethic groups using unpaired t test statistics, females native to savanna zone recorded higher mean left hand breadth than the Females native to forest zone, and the difference was statistically significant. Also, the mean difference in right hand breadths between the two groups showed statistical significance. However, the mean differences in left and right hand lengths, though were higher in the females native to savanna zone than females native to forest zone, were not significant. These present findings are in line with study findings reported in Nigeria between the Delta Igbos and Isokos where statistical significance were observed in the mean left and right hand breadths as well as the mean left hand lengths of both ethnic groups^10^. People from savanna zone of Ghana are noted for their physical activities, leafy diets and healthy cultural practices as well as the habitual locomotor and manipulative tasks using the hands such as weaving, smock and hat making etc. which contribute to the development of hand bones and muscles^21^, and this could explain the high hand dimensions observed for females native to savanna zone. The present findings also, contradict study report which found no statistical significance difference in hand dimensions when five ethnic groups were studied^16^.

Using binary logistic regression analysis, the left hand breadth was found to have shown to be better predictor of females native to savanna zone in this present study. However, the right hand breath and length as well as left hand length proved not to predict females native to forest zone. Therefore, only left hand breadth could exhibit potential in discriminating female populations native to these zones. Females native to forest zone had consistent prediction accuracy with all the hand dimensions (Table 5) although these predictive accuracies were lower than the 85% classification accuracy considered to be appropriate. This presupposes that the females native to forest zone may exhibit right hand dominance. Climate, nutrition and economic conditions between the savanna and forest zones in Ghana are quite different which may have influence on anthropometric development of hand, as climatic conditions and geographical locations have been reported to alter genetic differentiation within ethnic groups in Southern India^5^. Therefore, these could have significant contribution in predicting females native to these climatic zones using hand dimensions^5,22^.

## Conclusion

This present study observed significant differences in right and left hand breadth dimensions among the females native to forest and savanna zones where the females native to savanna zone recorded higher values than the females native to forest zone. A binary regression model also established that left hand breadth showed potential of predicting female population native of savanna zone with statistically significant level. The study therefore may speculate that left hand breadth could have the potential to differentiate female populations native to savanna zone from females native to forest zone in Ghana.

These study findings may play essential role in forensic identification during accidents or natural disasters, and also in ergonomics as well as workstations. Again, it is the first hand dimension study that considered same sex population (female) from different geographical zones in Ghana. Limitations to the study include the small sample data, restricted age range and also the inherent limitations associated with anthropometric studies. It is recommended that future studies should consider these same climatic zones but include large sample size of participants with comparison among specific age ranges. Future study should also perform cross-validation of the models that will be developed.

## Data Availability

The quantitative data used to support the findings of this study may be released upon reasonable application to the Head of Anatomy Department, School of Medicine and Dentistry, Kwame Nkrumah University of Science and Technology, who can be contacted at Telephone:: + 233 244 988328 and email: bimpongsamuel@gmail.com.

## Abbreviations

KNUST: Kwame Nkrumah University of Science and Technology
IBM: International Business Machines Corporation
SPSS: Statistical Package for Social Sciences
CHRPE: Committee on Human Research and Publications Ethics
EPV: Event per variable
LBH: Left hand breadth
RHB: Right hand breadth
LHL: Left hand length
RHL: Right hand breadth
SD: Standard deviation
